# Systemic Neuroprotection by Chlorogenic Acid: Antioxidant and Anti-inflammatory Evaluation in Early Neurodegeneration Induced by 3-Nitropropionic Acid in Mice

**DOI:** 10.1007/s11064-025-04356-4

**Published:** 2025-03-04

**Authors:** Angélica Cantero-Téllez, Leticia Moreno-Fierros, Gabriel Gutiérrez-Ospina, Ana Cecilia Santiago-Prieto, Imelda Juárez, Miriam Rodríguez-Sosa, Elizabeth Hernández-Echeagaray

**Affiliations:** 1 Facultad de Estudios Superiores Iztacala, Unidad de Investigación en Biomedicina, Universidad Nacional Autónoma de México, Av. de los Barrios #1, Los Reyes Iztacala, 54090 Tlalnepantla de Baz, Estado de México México; 2 Departamento de Biología Celular y Fisiología, Instituto de Investigaciones Biomédicas, Universidad Nacional Autónoma de México, Circuito, Mario de La Cueva S/N, C.U., Coyoacán, 04510 Ciudad de México, México; 3 Laboratorio de Anatomía Patológica, Hospital H+Querétaro, Privada Ignacio Zaragoza 16, Centro, 76000 Santiago de Querétaro, Qro Mexico; 4 Laboratorio de Neurofisiología del Desarrollo y la Neurodegeneración, UBIMED, FES-I, UNAM, Av. de los Barrios # 1, Los Reyes Iztacala, C.P.54090 Tlalnepantla de Baz, Estado de México, México

**Keywords:** Polyphenols, Neuroprotection, Mitochondrial dysfunction, Lipid peroxidation, Basal ganglia, Environmental toxins, Huntington’s disease

## Abstract

Neurodegeneration is characterized by the progressive loss of neurons commonly attributed to neurological causes. Studies published over the past two decades suggest that neurodegeneration may occur due to systemic diseases that compromise energy metabolism throughout the body. This metabolic imbalance develops over decades before neurodegeneration is clinically documented or inferred. It is now accepted that long-lasting oxidative stress and inflammation link neurodegeneration with altered energy metabolism in the body. Systemic prevention of these factors may reduce the odds of developing neurodegeneration and delay or prevent its progression as individuals age. Chlorogenic acid (CGA) is a polyphenol prevalent in fruits and vegetables that exhibits antioxidant and anti-inflammatory properties. It may serve as a systemic neuroprotectant when consumed regularly before the onset of neurodegeneration. To test this possibility, an experimental model of striatal early neurodegeneration induced by systemic administration of 3-nitropropionic acid (3-NP) was used. This toxin inhibits succinate dehydrogenase (SDH), disrupts electron flow and leads to increased production of reactive oxygen species (ROS) and a pro-inflammatory environment. The severity of symptoms induced by 3-NP varies depending on dosage, duration of exposure and administration route. In the brain, 3-NP affects striatal medium spiny neurons in the basal ganglia and in less degree pyramidal neurons from frontal cortex, a feature observed in Huntington’s disease (HD). The aim of this study was to investigate the antioxidant and anti-inflammatory properties of CGA in the 3-NP-induced model of early neurodegeneration. Systemic administration of CGA significantly reduced lipid peroxidation and promoted an anti-inflammatory profile in the brain when co-administered with 3-NP. These results support that CGA could serve as a systemic neuroprotectant in individuals challenged by environmental toxins that disrupt mitochondrial function.

**Graphical Abstract:**

Oxidative stress, induced in the striatum and frontal cortex by 3-NP treatment is avoided by CGA co-treatment, while the inflammatory response is relatively prevented in the 3-NP + CGA co-treatment.

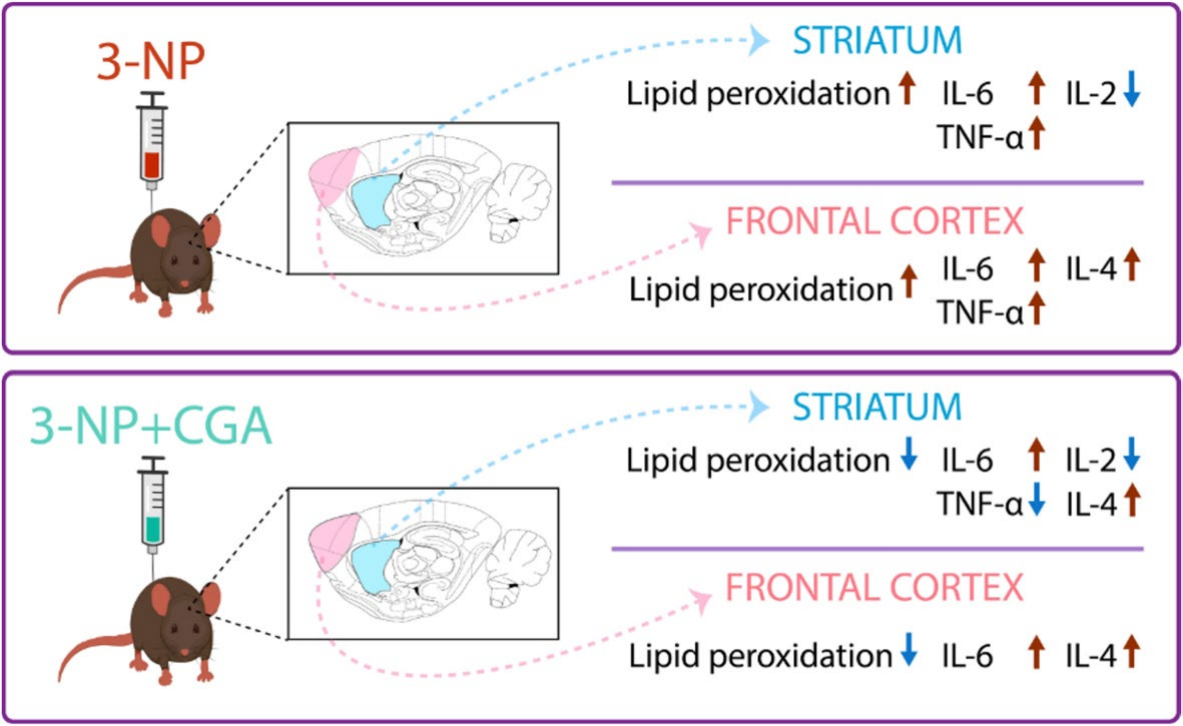

## Introduction

Neurodegeneration is a lifelong process that features not only the progressive loss of neurons in various regions across the central and peripheral nervous system, but a myriad of prodromic functional morphological disturbances that progress over decades before the pathognomonic neurological symptoms are identified [1]. Nowadays, neurodegeneration cannot be viewed as a pathological landmark of a neurological disorder, but as a long-term process that reflects a systemic dysregulation that involves, among others, chronic mitochondrial energy deficits leading to oxidative stress [2] and neuroinflammation. Preventing the occurrence of these factors might reduce the chances of developing and slow down/avoid the progression of neurodegeneration in susceptible individuals (e.g., Huntington’s disease, HD) or in those exposed to polluted environments (e.g., Idiopathic Parkinson’s disease, PD) [3].

CGA is a dietary polyphenol [4, 5], abundant in fruits and vegetables, and formed after the esterification of caffeic acid and quinic acid [6–8]. Studies have demonstrated that CGA is an effective antioxidant and anti-inflammatory agent in the brain of Swiss mice exposed to sodium arsenate [9]. We have also shown that CGA decreases systemic toxicity and genotoxicity in C57BL/6 mice treated with 3-NP [10] a mitochondrial toxin. The fact that in both cases CGA was administered systemically suggests that this compound may be used as a systemic neuroprotectant. Here, we tested whether the systemic administration of CGA prevents oxidative damage and neuroinflammation in the brain of mice treated with 3-NP, a mitochondrial toxin [11–13] found in legumes that induces degeneration of striatal medium spiny neurons [13–15] and motor dysfunction resembling HD, when ingested accidentally by livestock or humans [11]. 3-NP uncouples mitochondrial respiration by competitively inhibiting the succinate dehydrogenase (SDH) [16] which disrupts energy metabolism, resulting in increased oxidative stress [17] and inflammation [18, 19] across the body and brain (striatum and frontal cortex) of experimental animals [12, 20].

Our findings show that CGA reduces lipoperoxidation levels and proinflammatory cytokines, while increasing anti-inflammatory cytokines, without impacting liver function as indicated by transaminase levels.

## Methods and Materials

### Animals

Adult C57BL/6 male mice, born and raised in the in-home animal facility at the Facultad de Estudios Superiores-Iztacala (FES-I), Universidad Nacional Autónoma de México (UNAM). Mice were housed in plexiglass cages in groups of five and kept under a 12-h regular light/dark cycle at 25° ± 2 °C, having free access to food and water throughout the day. Mice were randomly divided into four groups (n = 5–8/group) and assigned to control group received vehicle (vhc; phosphate-buffered saline (PBS) (J.T. Baker®, # 3624–05), KH_2_PO_4_ (Sigma-Aldrich®, # P5655), KCl (CALEDON®, # 5920–1), Na_2_HPO_4_2H_2_O (Sigma-Aldrich®, # S0876)) pH 7.4 + 1% DMSO (Sigma-Aldrich®, #D4540). Those belonging to the reference group received CGA (100 mg/kg diluted in vhc, Sigma-Aldrich, #c3878). Mice that received 3-NP (15 mg/kg dissolved in the PBS, pH 7.2, Sigma-Aldrich®, # n5636) and neuro-protected mice (CGA + 3-NP) were administered CGA followed by 3-NP (15 mg/kg, see below) approximately 10 min later. CGA and 3-NP dosing are based on previous studies that looked for protective effects [10, 21]. 3-NP dosing/protocol leads to early striatal damage [13] without major motor deficits, except for clasping behavior, orofacial dyskinesia, and increased ambulatory activity [22], features also described in the early stages of other experimental animals that model HD (11). Every treatment lasted five days and was administered daily through intraperitoneal injections given at 12:00 PM.

It is important to note that 3-NP is one of several mitochondrial toxins commonly used to model neurodegeneration in experimental animals. Three of the most used are 3-NP, 1-methyl-4-phenyl-1,2,3,6-tetrahydropyridine (MPTP), and rotenone. While MPTP and rotenone uncouple the mitochondrial respiratory chain by interfering with complex I and damage mainly nigral dopaminergic neurons, 3-NP blocks complex II and harms striatal medium spiny neurons. This renders MPTP and rotenone suitable to model some aspects of PD, and 3-NP apt to model HD [3, 12, 23].

Animal procedures were approved by the ethics committee of FESI-1242, UNAM. Every procedure followed the Norma Oficial Mexicana NOM-062-ZOO-1999 that sets the standards for the production, care, and use of laboratory animals in Mexico (https://www.gob.mx/senasica/documentos/nom-062-zoo-1999).

### Brain Sampling

Mice were euthanized with ether (J.T. Baker, #: 9340–02) eight days after initiating the treatment. The corpses were decapitated, and the brains rapidly extracted from the skull. The striatum and frontal cortices were dissected, and the samples weighted and sonicated in lysis buffer (TRIS (USB®, # 75825), Triton (Sigma-Aldrich®, # 9002-93-1), Glycerol (Sigma-Aldrich®, # G5516) and proteases inhibitor (Roche® completeMini EDTA-free, #11,836,170,001) during 20 s at 4 °C temperature. The tissue samples were further centrifuged (4200 rpm) for 10 min at room temperature. Supernatants were collected in microfuge tubes and stored at − 80 °C until used. Later, these samples were used to estimate lipoperoxidation (LPO) and the concentration of pro- and anti-inflammatory cytokines. Protein estimation was carried out with Bradford method (Bio Rad No. 500–0205).

### Liver Histological Analysis

Livers were dissected from the mouse corpses after euthanasia. Once freed, the livers were weighed and a tissue slab (0.5 cm thick) traversing the entire transverse plane of each liver was obtained and fixed by immersion in buffered paraformaldehyde (diluted 4% in phosphate buffer 0.1 M, 7.4 pH) for 72 h at room temperature. After fixation, the tissue slabs included in paraffin by using automated standard protocol (Shandon Citadel ST1075 05 M, Reichert Jung 2030 113,778; today’s Reichert A Metek, NY, USA). Once included, tissue samples were sectioned (2μm thick) with aid of a microtome and collected on to poly-L-lysine coated glass slides. Tissue sections were then dewaxed, re-hydrated and stained with Harris’ hematoxylin and eosin stain. Finally, tissue sections were dehydrated through an alcohol gradient, cleared in xylene and cover-slipped with a synthetic mounting resin (Hycel Reactivos Químicos, Zapopan, Jalisco, Mx).

The histological material was observed blind to the experimental condition by an experienced pathologist who searched for signs of hepatic cytotoxicity. The analysis focused on perivenular hepatocytes because they perform primary detoxification functions and suffer first and greatly when there is severe intoxication. So, the sections were scanned to locate hepatic lobules in which the central venules were structurally intact. Once identified, the presence of fibrosis, necrosis and leukocyte infiltration in the interstitial space, and signs of hepatocyte atrophy, vacuolar degeneration and autolysis were searched for. All observations were conducted by using a bright field Olympus BX40 microscope (400 × 0.62 mm).

### Estimating Transaminases Activity in Serum

Blood samples were collected during the exsanguination process that occurred after decapitation. To avoid hemolysis, the samples were collected by dropping the blood into microfuge tubes; they were allowed to coagulate for 15 min while in ice. The tubes were then centrifuged at 2000 rpm for 5 min at 4 °C. The serum was collected and stored at − 80 °C until used.

The activity of alanine aminotransferase (ALT; also referred as to glutamic pyruvic transaminase) and aspartate aminotransferase (AST; also known as glutamic oxaloacetic transaminase) was estimated with the assistance of colorimetric assay kits according to the supplier’s instructions (AST-activity quantitative assay, # MI41264); ALT-activity quantitative determination assay, # SP41274; SPINREACT, Barcelona, Spain). In both cases, the activity of the corresponding transaminases is proportional to the amount of oxaloacetate or pyruvate formed within a predetermined time. This is measured by the reaction of these compounds with 2,4-dinitrophenylhydrazine (DNPH) in an alkaline medium, which forms a colored hydrazone. Light absorbance values for each metabolite were estimated by using a spectrophotometer (Epoch BioTeK® Instruments, Inc. 2011. USA) at 505λ. Finally, absorbance values were extrapolated onto a calibration curve to determine the rate of substrate consumption, the latter expressed as micromoles per min for 5 min under standard conditions (vendor instructions, BEIS45-E 8/01/16).

### Estimating Lipid Peroxidation

Lipid peroxidation (LPO) was estimated by quantifying malondialdehyde (MDA), a product of lipid peroxidation of polyunsaturated fatty acids. A common way to estimate MDA is by reacting it with thiobarbituric acid (TBA) under acidic conditions to form thiobarbituric acid reactive substances (TBARS). The LPO Assay Kit used in this work (Sigma, # MAK085) estimates MDA concentration by quantifying TBARS colorimetrically (absorption = 532λ).

### Assessing Neuro-Inflammation

To assess the brain inflammatory response, we measured the concentrations of four key cytokines (TNF-α, IL-6, IL-2, IL-4) which have been implied in neurodegenerative process, as proinflammatory (TNF-α, IL-6), and as anti-inflammatory (IL-4, IL-2) cytokines in supernatants obtained from the frontal cortex and striatum using standardized ELISAs following the manufacturer’s instructions (Murine Standard ABTS ELISA Development kits for TNF-α # 900-K54, IL-6 # 900-K50, IL-2 # 900-K108, and IL-4 # 900-K49, Peprotech®, NJ, USA). Briefly, reconstituted aliquots of capture and detection antibodies, cytokines biotin-HRP conjugates (5.5 μL) were stored at − 70 °C. The day of the experiment, the 96-well plate was coated with capture antibodies (1 μg /μL) overnight at 4 °C. The wells were washed (0.05% Tween-20 in PBS) and blocked (1% BSA in PBS) for an hour at room temperature. After washing, supernatant was added to Wells (dilution 1:3 in PBS, 40 μg of total protein by well) and incubated overnight at 4 °C. After washing, wells were coated with detection antibodies (TNF-α, 0.5 μg /mL; IL-6, 1 μg/mL; IL-2, 0.25 μg /mL and IL-4, 1 μg /mL) and incubated for 2 h at room temperature. After further washing, the biotin-HRP conjugate was added and incubated for 30 min at room temperature.

Finally, ABTS with 30% hydrogen peroxide was added, the plate was covered and incubated for 5 min. The plate was read at 405 λ every 5 min for a total of 50 min. A standard curve was established with serial dilutions for IL-6, IL-4, IL-2, and TNF-α, followed by a 2-h incubation.

### Data Analysis

The statistical design for body weight included two factors: (A) Treatments (4 experimental groups) and B) Days of Treatment (5 days). Hence, we statistically analyzed the effect that each treatment had on the body weight of the specimens of each experimental group by daily weighting the mice during five days (repeated measures). Because body weight data distribution passed the D’Agostino & Pearson normality test (α = 0.05), this database was analyzed by using a parametric two-way, repeated measures Analysis of Variance (ANOVA). Significant differences between groups were uncovered by using Tukey´s *post-hoc* test. Differences were considered significant at p < 05.

In contrast to body weight, liver weight was measured once at the end of the experiment. The statistical design used to evaluate differences among groups was selected after evaluating the data distribution using the Shapiro–Wilk normality test (α = 0.05). Since the data distribution was not normal, we analyzed the liver weight data set using a Kruskal–Wallis ANOVA on Ranks. Significant differences between groups were revealed using Dunn’s *post-hoc* test. Differences were considered significant at p < 0.05.

Similarly to liver weight, serum transaminase activity, brain LPO, and brain cytokine concentrations were quantified at the end of the experiments. None of these data sets passed their respective normality tests (for serum transaminase activity: Kolmogorov–Smirnov normality test (α = 0.05); for brain LPO and brain cytokine concentrations: Shapiro–Wilk normality test for TNF-α and IL-6, (α = 0.05); Kolmogorov–Smirnov normality test for IL-2 and IL-4, (α = 0.05). Consequently, we used Kruskal–Wallis ANOVA on Ranks to statistically analyze the data sets from these parameters. For these parameters, except for serum transaminase activity where no differences were noted between groups, significant differences between groups were assessed using Dunn’s post-hoc test and considered significant at p < 0.05. Databases were organized and analyzed using GraphPad Prims 7.00 Software ©.

## Results

### Body Weight

**Fig. 1:**
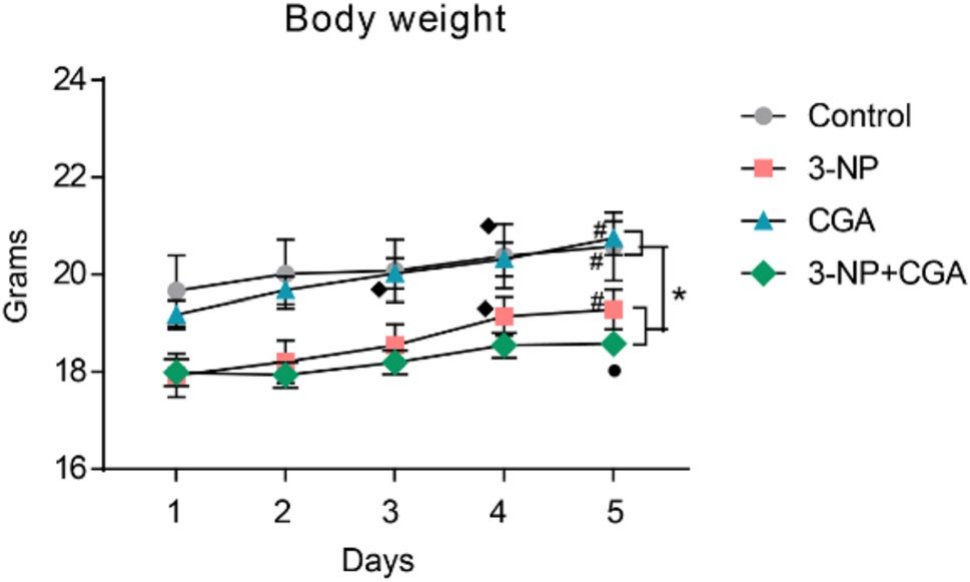
Body weight. Body weight of C57BL/6 mice vehicle treated (control, grey, n = 23), 3-NP (pink, n = 23), CGA (blue, n = 23) and 3-NP + CGA (green, n = 23). Means are shown, ± standard errors. Two-way repeated measures ANOVA and Tukey’s post hoc test were performed. * p < 0.05 vs control and CGA. p < 0.05 vs 3-NP. day when body weight increase started. # p < 0.0001, day 5 vs day 1

Weight loss increased morbidity and mortality in patients undergoing neurodegenerative processes [24]. Fair enough, the two-way repeated measures ANOVA for the body weight of Control, 3-NP, CGA, or 3-NP + CGA treated mice revealed a statistically significant effect of the treatments (*F*_*3, 66*_ = 4.883, p = 0.0039), a significant effect of time (days, *F*_*4, 88*_ = 33.76, p < 0.0001), and a significant interaction between the treatments and time (*F*_*12, 264*_ = 2.105, p = 0.017; Fig. 1). In brief, body weight was similar between the control and CGA groups, as well as between the 3-NP and 3-NP + CGA groups. However, significant differences were observed between these groups over time. So, 3-NP indeed interferes with body weight gain.

### Liver Functional Morphology

**Fig. 2:**
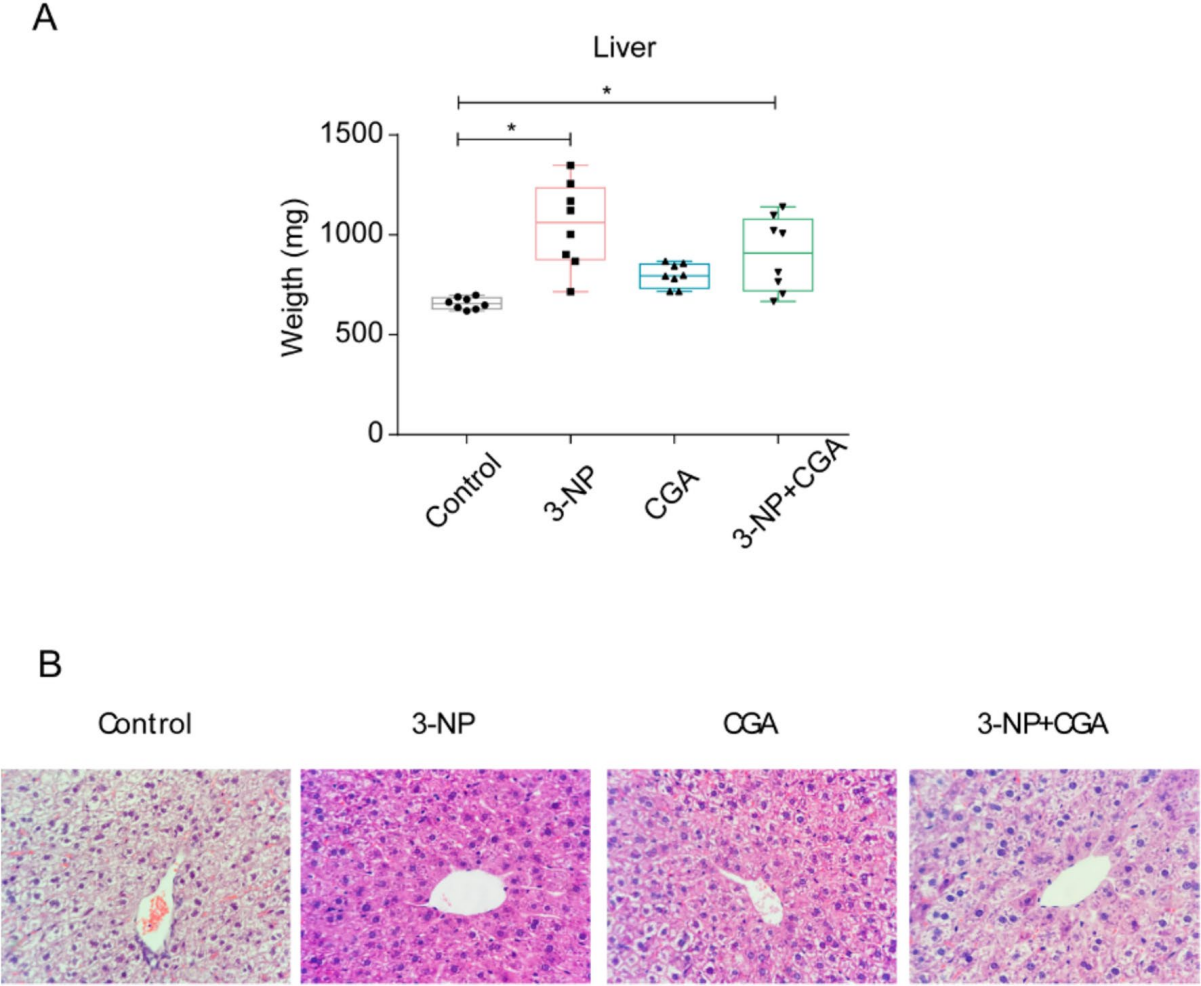
Liver weight. **A** Liver weight of C57BL/6 mice vehicle treated (control, gray), 3-NP (pink), CGA (blue) and 3-NP + CGA (green). Medians are shown with interquartile ranges. Kruskal–Wallis test with Dunn’s post hoc test was performed. * p < 0.05, 3-NP and 3-NP + CGA vs control, n = 8 per group. **B** Histopathological analysis of representative mouse liver samples stained with H&E revealed no significant structural changes among the groups

Recent research indicates that altered communication between the liver and the brain contributes to neurodegeneration [25]. Given the liver’s role in primary detoxification and the potential damage toxicants can cause to its functional morphology, it was important to verify its anatomical and functional integrity under the present experimental conditions. Qualitatively, the dataset analyzed with Kruskal–Wallis ANOVA on Ranks (*H*_*3*_ = 19.805, p < 0.001) followed by Dunn’s post-hoc (p < 0.05) tests revealed that liver weight was similar between the control and CGA groups, however significant differences were found between the control and 3-NP groups and the control and 3-NP + CGA groups (Fig. 2A). Notably, 3-NP + CGA cotreatment tended to prevent the increment in liver weight.

Interestingly, liver weight variability increased following all treatments, with the greatest diversity observed in mice treated with 3-NP, followed by those administered 3-NP and CGA (Fig. 2A). This observation indicates that the response to 3-NP and to 3-NP + CGA treatments is idiosyncratic rather than categorical. The reasons for this response are unclear, but it may result from differences in individual baseline metabolic status and antioxidant defenses across the mice population before the experiment started.

Heavier livers in numerous 3-NP and 3-NP + CGA treated mice might imply shifts in the cytoarchitectural organization of the organ. We evaluated whether this was indeed the case. Livers from all mouse groups exhibited some degree of perivenular hepatocyte vacuolar degeneration and autolysis, changes commonly related to inadequate fixation. Aside from this artifact, the histological survey conducted in liver samples from all experimental groups showed well-preserved perivenular hepatic cytoarchitecture (Fig. 2B). Hence, the 3-NP administration protocol used did not seem to damage the liver, under the doses and days of treatment used in this study.

**Fig. 3:**
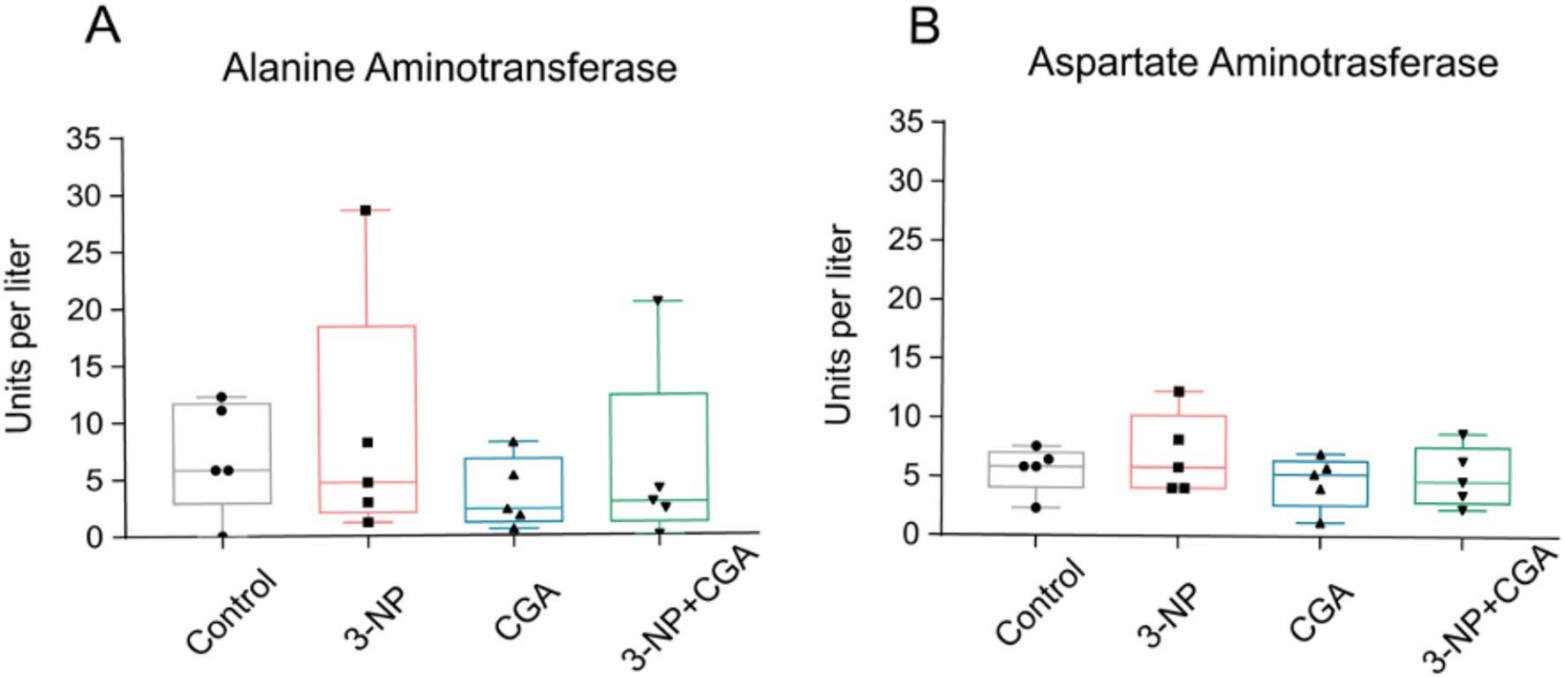
Hepatic transaminases. Hepatic transaminases in the serum of C57BL/6 mice vehicle treated (control, grey), 3-NP (pink), CGA (blue) and 3-NP + CGA (green). Medians (with interquartile ranges) of alanine aminotransferase (**A**) and aspartate aminotransferase (**B**) in the serum of C57BL/6 mice. No statistical significance was observed (Kruskal–Wallis test with Dunn’s post hoc test was performed). n = 5 per group

Finally, an indirect way to assess liver functional integrity is by examining changes in the activity of circulating hepatic enzymes, as these enzymes levels increase following hepatic damage. Accordingly, we estimated ALT and AST levels in the serum obtained from control, 3-NP, CGA, and NP + CGA treated mice. As shown in Fig. 3, the Kruskal–Wallis ANOVA on Ranks indicated no significant differences between experimental groups (AST, *H*_*3*_ = 0.972, p = 0.80; ALT, *H*_*3*_ = 1.92, p = 0.58). These results align with the histological findings, as they also suggest that no hepatic damage was induced by 3-NP administration.

### Brain Lipid Peroxidation

**Fig. 4:**
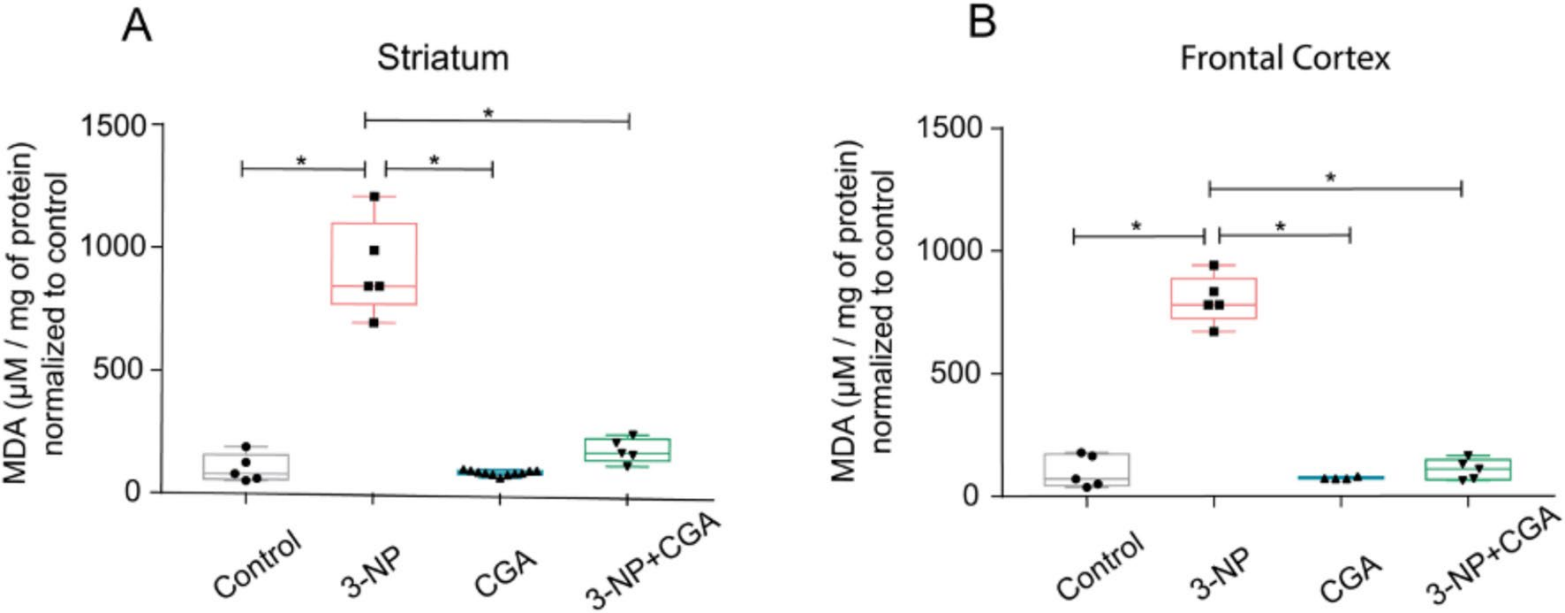
Lipid peroxidation. Medians (with interquartile ranges) of lipid peroxidation levels in **A**) striatum and **B**) frontal cortex of C57BL/6 mice treated with vehicle (control, grey), 3-NP (pink), CGA (blue) and 3-NP + CGA (green). Kruskal–Wallis test with Dunn’s post hoc test was performed, showed differences between 3-NP vs control, CGA and 3-NP + CGA vs 3-NP, * p < 0.05, n = 5 per group

The reduction of body weight gain might indicate that 3-NP injures the brain secondary to systemic effects through local mechanisms of ROS production and oxidative stress [12]. To strengthen this view, we estimated LPO in the striatum and frontal cortex of control mice and those administered with 3-NP, CGA and 3-NP + CGA (Fig. 4). Qualitatively, LPO was similarly low in the striatum and frontal cortex of control mice and those administered CGA and 3-NP + CGA. In sharp contrast, LPO was significantly greater in 3-NP treated mice as compared to the other groups (Fig. 4). The most significant result, though, was that CGA prevented 3-NP-induced lipid peroxidation in both the striatum (*H*_*3*_ = 17.509, p < 0.001) and frontal cortex (*H*_*3*_ = 10.692, p = 0.014).

### Brain Cytokine Concentration

**Fig. 5:**
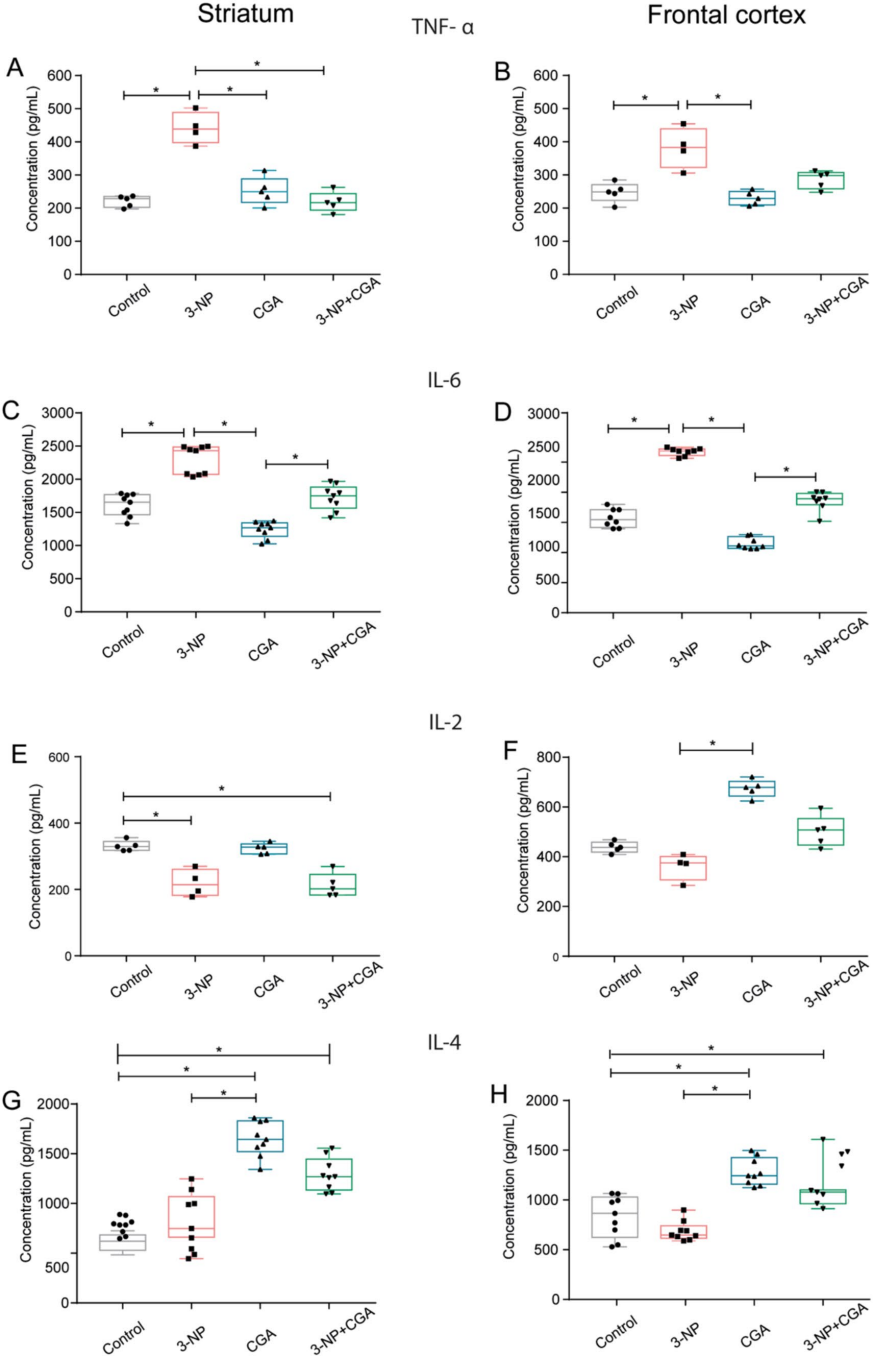
Cytokines levels. Levels of pro- and anti-inflammatory cytokines. Medians (with interquartile ranges) of concentrations of TNF-α (**A** and **B**, n = 5), IL-6 (**C** and **D**, n = 8), IL-2 (**E** and **F**, n = 5), and IL-4 (**G** and **H**, n = 8) in the striatum and frontal cortex of of C57BL/6 mice treated vehicle (control, grey), 3-NP (pink), CGA (blue) and 3-NP + CGA (green). Kruskal–Wallis test with Dunn’s post hoc test was performed. * p < 0.05

Inflammation plays a central role in the etiopathogenesis of neurodegeneration. CGA, on the other hand, reduces systemic inflammation and neuroinflammation in a variety of mouse models [26–28]. We then evaluated whether the systemic administration of 3-NP increased inflammation and whether the systemic protection provided by CGA was able to prevent it by studying the concentrations of proinflammatory (TNF-α and IL-6), anti-inflammatory (IL-4), and state-dependent dual (IL-2) cytokines in the mouse striatum and frontal cortex. Accordingly, we found elevated concentrations of TNF-α and IL-6 in the striatum and frontal cortex of 3-NP treated mice compared to their control counterparts. These differences were significant for TNF-α in the striatum (*H*_*3*_ = 10.680, p = 0.014) and frontal cortex (*H*_*3*_ = 12.911, p = 0.005), as well as for IL-6 in the striatum (*H*_*3*_ = 29.494, p = 0.0001) and frontal cortex (*H*_*3*_ = 28.253, p = 0.001). As predicted, the concentrations of TNF-α and IL-6 in both brain structures were kept within the control range in CGA and, to some extent, in 3-NP + CGA treated mice for TNF- α but not for IL-6 which showed a non-significant reduction. These results suggest that, under unchallenged conditions, CGA helps stabilize neuroinflammation at a low level, but when the mouse is co-treated with 3-NP + CGA, it tends to reduce it (Fig. 5).

With respect to the anti-inflammatory IL-4 and the state dependent dual IL-2, we found significant differences for IL-2 in the striatum (*H*_*3*_ = 13.770, p = 0.003) and the frontal cortex (*H*_*3*_ = 15.834, p = 0.001) and for IL-4 in the striatum (*H*_*3*_ = 28.143, p = 0.001) and the frontal cortex (*H*_*3*_ = 26.09, p = 0.001). Dunn’s multiple comparison analysis revealed that IL-2 significantly decreased in the striatum of the 3-NP treated mice compared to the control group (p < 0.05), but the cotreatment did not revert IL-2 reduction. Conversely, IL-2 increased notably in the frontal cortex of mice treated with CGA relative those administered 3-NP (p < 0.05) and the cotreatment 3-NP + CGA prevents its reduction. Lastly, IL-4 concentrations in the striatum and frontal cortex increased significantly (p < 0.05) in mice administered CGA or 3-NP + CGA compared to control mice or to those administered 3-NP. These findings indicate that CGA may shift the balance of neuroinflammation towards an anti-inflammatory profile differentially across brain regions, highlighting the potential benefits of regular CGA consumption.

## Discussion

Neurodegeneration is an end-stage result of chronic mitochondrial energy deficits, causing increased oxidative stress and neuroinflammation in the brain. Systemic treatments that prevent these factors may reduce the risk of developing or slow the progression of neurodegeneration. Based on this premise, in this work, we assessed whether the systemic administration of CGA, a polyphenol found in a variety of comestible nuts, vegetables and seeds, protects the striatum and the frontal cortex from damage induced by 3-NP, a compound that interferes with the mitochondrial respiratory chain. 3-NP is used to mimic the mitochondrial dysfunction and neuro-histopathology observed in HD patients in experimental animal models. In rodents, the severity of neurodegeneration induced by 3-NP depends on the species/strain, as well as on the concentration, frequency, and duration of 3-NP administration [11, 14]. Under the protocol used here, we modeled early, presymptomatic stages of HD, to investigate the neuroprotective effects of CGA on the frontal cortex and striatum, which are functionally impacted in HD. Our findings support that systemic CGA may help to protect the brain in the early stages of HD because, in mice administered 3-NP + CGA, the compound significantly reduced oxidative stress and shifted the balance of the immune response towards an anti-inflammatory. The effect appears to be brain-specific under our experimental conditions, as CGA did not improve weight gain over time in mice also treated with 3-NP (Fig. 1). This is a systemic response commonly observed in mice treated with 3-NP while modeling HD presymptomatic stages [29, 30].

Mice treated with 3-NP showed an increase in liver weight compared to the control group (Fig. 2A), consistent with previous studies [31, 32]. These findings suggest that 3-NP treatment has systemic effects, potentially affecting liver physiology and inducing stress or inflammation. To further explore these effects, we examined liver histology and measured ALT and AST levels, which are commonly used as markers of liver function [33]. However, we observed no significant morphological changes (Fig. 2B) or alterations in liver transaminase levels (Fig. 3). This lack of substantial changes in these enzymes indicates that, at the time point measured, 3-NP treatment did not result in significant functional damage or impairment of liver function. This statement is supported for the lack of structural damage in hepatic tissue. Further investigation is needed to understand liver atrophy produced by 3-NP treatment.

But how does the systemic administration of CGA counteract oxidative stress arising from 3-NP induced mitochondrial respiration uncoupling? Polyunsaturated fatty acids are key targets of oxidative radicals when in excess. Therefore, measuring LPO is a useful tool to assess CGA’s ability to protect the brain from oxidative damage induced by 3-NP. This is precisely what our experiments evidenced. Indeed, the striatum and frontal cortex of mice treated with 3-NP + CGA displayed LPO levels comparable to those observed in control and CGA treated mice, and far less LPO levels than those documented for 3-NP treated mice (Fig. 4A, B). Hence, CGA associated neuroprotection comes from its ability to decrease local oxidative stress. In this regard, it is known that CGA scavenges hydroxyl radicals (OH), peroxide radicals (OOH), and superoxide ions (O_2_¯) in a dose-dependent manner [6–8, 34]. It also activates the Nrf2 pathway, which promotes the upregulation of antioxidant enzymes, helping to maintain mitochondrial function and integrity, thereby preventing mitochondrial dysfunction and excessive ROS production [35]. These properties are also shared by other polyphenols, such as Butin [36], Silymarin [37, 38], and Rutin [39], when assessed for their protective effects in the 3-NP degeneration model in rats.

Oxidative damage throughout the body triggers inflammation when Damage-Associated Molecular Patterns (DAMPs) are released by stressed or dying cells, a process that also occurs in the case of 3-NP, as demonstrated in our study. It is likely that CGA may reduce brain inflammation by both indirectly lowering local oxidative stress and directly modulating immune cell function [40]. Cytokines are immune messengers released by immune cells to coordinate immune efforts. The elevations, falls, or stability of key cytokine concentrations, even if not all reach statistical significance, can inform us about the chemical code used by the immune system to bias its response while confronting the challenges it faces [41].

We then estimated the proinflammatory cytokines TNF-α and IL-6 in the striatum and frontal cortex of control mice and those administered with 3-NP, CGA, or 3-NP + CGA. As expected, both cytokines were found to be significantly increased in both brain regions of 3-NP-treated mice compared to control and CGA-treated mice, indicating that an inflammatory response was induced (Fig. 5A–D). This is not surprising, given that TNF-α contributes to the development and progression of neurodegenerative diseases [42–48], by activating inflammatory and apoptotic signaling pathways [43, 44], which exacerbates disease progression and neuronal loss [45–47]. Our results also support that CGA a phytoantioxidants can reduce levels of TNF-α [48–50].

A similar pattern was observed for IL-6, whose increased availability exacerbates neurodegenerative conditions [51–55], despite its neuroprotective role at lower concentrations [55]. IL-6 plays both neuroprotective and inflammatory roles [55]; while IL-6 can support neuronal regeneration and oligodendrocyte differentiation, its overproduction contributes to chronic inflammation and oxidative stress, exacerbating neurodegenerative conditions [51–54, 56, 57]. Indeed, IL-6 elevation levels have been observed in patients afflicted by HD [58]. In our study, CGA + 3-NP cotreatment did not reduce IL-6 levels significantly compared to the 3-NP-treated group. These findings suggest that while CGA may tend to modulate IL-6 levels, it may need more days of CGA treatment to reach statistical significance in the context of this HD model.

Nonetheless, CGA alone or when co-administered with 3-NP, maintained TNF-α and IL-6 levels at or near those observed in control mice. This finding supports that CGA, in addition to its anti-inflammatory actions facilitated by its antioxidant properties [34, 35], also possesses inherent anti-inflammatory effects which may influence the regulation of inflammatory mediators involved in the pathology of neurodegeneration. In support of this argument, other experiments have shown CGA suppresses key inflammatory signaling pathways [26–28, 48, 49],

Notably, Mitochondrial inhibition induced by 3-NP increased IL-6 production, like mechanisms observed in HD [59, 60]. Interestingly, CGA has been shown to prevent the decline in mitochondrial complex activity and attenuate IL-6-related inflammatory processes in a PD model [61], further experiments should investigate if CGA modulates mitochondrial alteration produced by 3-NP in the context of HD.

Additionally, our findings concerning to IL-2 and IL-4 revealed nuanced effects. IL-2 levels decreased in the striatum of the 3-NP-treated group were not restored by CGA treatment (Fig. 5E). This observation contrasts with reports of elevated IL-2 levels in PD patients [62, 63] and emphasizes the complex role of IL-2 in immune regulation and neuronal trophic support [64–66]. The group treated only with CGA showed no changes in IL-2 expression compared to the control group in the striatum; but CGA increased IL-2 in frontal cortex (Fig. 5F). The lack of effect on IL-2 is consistent with previous reports [67], which found that CGA did not affect IL-2 levels but instead promoted an increase in IL-4 (see below).

A study found that HD patients with severe motor and non-motor symptoms (excluding chorea) had higher IL-2 levels compared to those with milder symptoms [68], suggesting that IL-2 elevation is linked to disease severity, which could explain the lack of CGA’s effect on IL-2 in our early-stage HD model. IL-2 is a cytokine with both pro-inflammatory and anti-inflammatory effects. It promotes lymphocyte proliferation, which can drive inflammation, but also stimulates regulatory T cells (Tregs) to suppress inflammation by inhibiting M1 macrophages and reducing Th17 cell activity. In the context of neural inflammation, IL-2 helps to balance Th17 and Tregs, offering potential therapeutic benefits for neuropathy and autoimmune neuroinflammation by reducing inflammation [69]. The reduced IL-2 expression in the 3-NP treatment group suggests that 3-NP may impair Treg cell differentiation, probably promoting inflammation and autoimmunity, common in neurodegeneration. Notably, CGA treatment alone does not stimulate IL-2 production, and co-treatment with 3-NP and CGA does not restore IL-2 levels in the striatum. Thus, CGA’s effect in reducing disease severity appears to be unrelated to Treg differentiation in the striatum but may involve the cortex.

Although research on IL-2′s role in neurodegenerative diseases is limited, studies [70, 71] suggest it may offer therapeutic benefits in other neurodegenerative conditions like Alzheimer’s disease (AD), where IL-2 increased Treg cells in the brain, improved memory, restored spinal density, and reduced amyloid plaques [72].

On the other hand, IL-4 levels increased in the striatum and frontal cortex of the CGA and CGA + 3-NP groups. IL-4, is known for its anti-inflammatory properties, helps to inhibit macrophage activation, promotes neuronal repair, and regulates local immune responses [73–76]. IL-4 is a Type 2 T-helper cytokine that supports the pro-regenerative Type 2 immune response, primarily mediated by Type 2 T-helper cells and M2 macrophages. This response helps resolve inflammation, promoting nerve regeneration and functional recovery [74].

The lack of a significant increase in IL-4 in the 3-NP-treated group contrasts with findings in advanced HD, where IL-4 levels are typically elevated [77–79]. IL-4 is typically expressed during adaptive immunity, and since our model replicates the early stages of HD, this suggests that adaptive immunity may not yet be fully activated. However, the increase in IL-4 observed in both the striatum and cortex of the CGA-treated group indicates that CGA may promote an anti-inflammatory environment, which could support neuronal regeneration and reduce neuroinflammation (Fig. 5G–E). Also, IL-4 increase in the co-treatment group (3-NP + CGA), may be due to the ability of CGA to reduce ROS [34], which drive the production of pro-inflammatory cytokines.

It is important to understand that, to reduce inflammation induced by 3-NP, CGA should target the immune cells of the nervous system, specifically microglia and astrocytes. [75]. Research has shown that 3-NP induces astrogliosis and activates microglia through the production of ROS [80, 81]. Upon activation, microglia polarize into M1 and M2 phenotypes [75]; M1 is proinflammatory, producing cytokines like TNF-α and IL-6, while M2 is anti-inflammatory, producing cytokines such as IL-4 and IL-10 which counteract M1’s effects and protects neurons [27]. CGA appears to modulate this polarization, inhibiting M1 activation and promoting M2 polarization, thus reducing inflammation and neuronal damage [27].

In summary, CGA treatment mitigated the inflammation and oxidative stress induced by 3-NP. Although our study focuses on effects of CGA on early stages of 3-NP-induced neurodegeneration and short-term interventions may not be representative of the chronic or long-term effects of 3-NP and CGA administration. Our findings emphasize the potential of CGA as a therapeutic agent for prevention or retardation of neural degeneration in addition reducing inflammation, CGA has neuroprotective properties, that makes CGA a particularly attractive candidate for treating neurodegenerative diseases, where oxidative stress, inflammation and neuronal loss are significant concerns. However, future studies should focus on strategies to enhance CGA bioavailability. Its bioavailability, which is influenced by factors such as intestinal absorption [82], hepatic metabolism, transformation by intestinal microbiota, and the method of consumption [83], may limit the amount of CGA entering the bloodstream, potentially reducing its biological and pharmacological efficacy [8]. Therefore, further research is needed to optimize its bioavailability before advancing translational medicine and improving preclinical and clinical studies in humans [84].

## Data Availability

All data from this study will be available upon request to the corresponding author.
